# Multivariate evaluation of the effectiveness of treatment efficacy of cypermethrin against sea lice (*Lepeophtheirus salmonis*) in Atlantic salmon (*Salmo salar*)

**DOI:** 10.1186/1746-6148-9-258

**Published:** 2013-12-20

**Authors:** Daniel F Jimenez, Crawford W Revie, Simon P Hardy, Peder A Jansen, George Gettinby

**Affiliations:** 1 Norwegian Veterinary Institute, PO Box 50 Sentrum, 0106 Oslo, Norway; 2 Atlantic Veterinary College, University of Prince Edward Island, Charlottetown, Canada; 3 University of Strathclyde, Glasgow, Scotland, UK

**Keywords:** *Lepeophtheirus salmonis*, Atlantic salmon, Treatment effectiveness, Multivariate analysis

## Abstract

**Background:**

The sea louse *Lepeophtheirus salmonis* is the most important ectoparasite of farmed Atlantic salmon (*Salmo salar*) in Norwegian aquaculture. Control of sea lice is primarily dependent on the use of delousing chemotherapeutants, which are both expensive and toxic to other wildlife. The method most commonly used for monitoring treatment effectiveness relies on measuring the percentage reduction in the mobile stages of *Lepeophtheirus salmonis* only. However, this does not account for changes in the other sea lice stages and may result in misleading or incomplete interpretation regarding the effectiveness of treatment. With the aim of improving the evaluation of delousing treatments, we explored multivariate analyses of bath treatments using the topical pyrethroid, cypermethrin, in salmon pens at five Norwegian production sites.

**Results:**

Conventional univariate analysis indicated reductions of over 90% in mobile stages at all sites. In contrast, multivariate analyses indicated differing treatment effectiveness between sites (p-value < 0.01) based on changes in the proportion and abundance of the chalimus and PAAM (pre-adult and adult males) stages. Low water temperatures and shortened intervals between sampling after treatment may account for the differences in the composition of chalimus and PAAM stage groups following treatment. Using multivariate analysis, such factors could be separated from those which were attributable to inadequate treatment or chemotherapeutant failure.

**Conclusions:**

Multivariate analyses for evaluation of treatment effectiveness against multiple life cycle stages of *L. salmonis* yield additional information beyond that derivable from univariate methods. This can aid in the identification of causes of apparent treatment failure in salmon aquaculture.

## Background

*Lepeophtheirus salmonis* is a major ectoparasite pathogen of farmed Atlantic salmon (*Salmo salar*) in the Northern Hemisphere. Sea lice infestations have a detrimental effect on health, production and market value of fish. The ecological impact on intensive salmon aquaculture has been of concern since published reports first suggested a link between the decline of wild salmon stocks and the presence of sea lice on salmon farms [1-6]. The Norwegian Food and Safety Authority (NSFA) have implemented precautionary measures to reduce the impact of sea lice from salmon production sites. These measures mandate the delousing or harvesting of production sites once sea lice infestations surpass the threshold set by NSFA [7,8]. While novel intervention strategies are being explored, the successful control of sea lice infestations in production sites is currently heavily dependent on effective delousing using chemotherapeutants.

Management systems for production of salmon in Norway have rapidly grown in the last decade, largely through intensification of fish production through the use of large pens and automation of feeding. Such operations however may magnify certain problems associated with the application of bath treatments. For example, the effectiveness of topical bath administrations is dependent on accurate dosage and a rapid and uniform distribution of the chemotherapeutant in the water column [9]. Inadequate medicinal exposure will result in incomplete treatment. The unintended effects of inadequate treatments include the repeated usage of chemotherapeutants over the production cycle and a risk that chemical resistance will emerge in sea lice populations [10].

Evaluation of field treatment effectiveness should identify those treatments that do not achieve the expected effect. Whilst reported treatment failures have been identified in Norway using alternative methods such as bioassays and probit modelling [11], current methods monitor the average reduction in mobiles (pre-adult and adult sea lice) following treatment. This simplified analysis risks overlooking changes that may cause treatment failure such as reduced sensitivity to chemotherapeutants.

Multivariate analyses enable the concurrent evaluation of effectiveness of treatment against all sea lice life cycle stages. Delousing effectiveness is multifactorial, being dependent on the interaction between biotic factors, such as life cycle stage, gender and phase in the molting cycle, as well as abiotic factors such as water temperatures and salinity. Consequently, a more robust interpretation of treatment effect can be made by incorporating data from sea lice life cycle stages. This type of approach is used in ecological studies to evaluate the effect or alterations that environmental stressors or toxicants produce in the composition of species community [12,13].

Our aim was to develop improved methods for the evaluation of treatment effectiveness in the field. Evaluation of treatment effectiveness against stages of salmon lice using multivariate methods provides a complete assessment of the overall effect of the drug and allows detailed comparisons between treatments. Specifically, it reveals those life cycle stages of *L. salmonis* that after treatment were characteristic of particular sites and hence which had potential use as indicators of treatment failure. Multivariate analysis also reveals other aspects that can be of interest when evaluating drug effects such as the changes in composition of *L. salmonis* stage groups following treatment.

## Results

### Presence and abundance of sea lice by stage group

**Figure 1:**
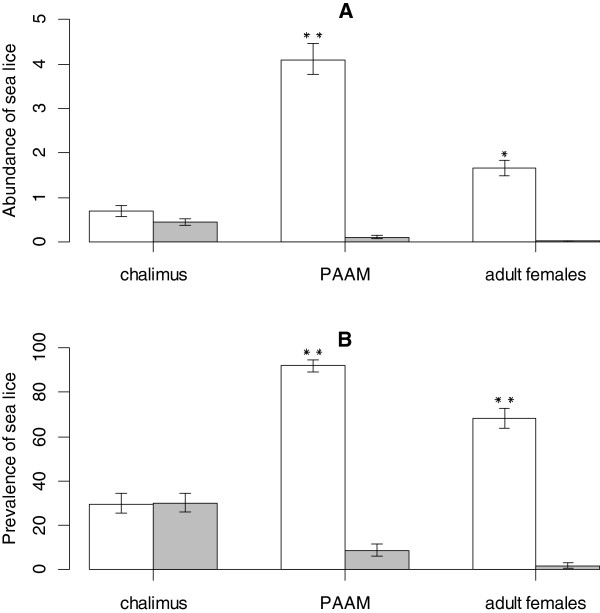
**Prevalence and abundance of sea lice by stage group before and after treatment. (A)** Mean abundance and **(B)** prevalence values for chalimus, PAAM and adult female stages before (open box) and after (shaded box) treatment with cypermethrin. In total, 33 treatments were performed at five different production sites between November 2011 and February 2012. Significance values: 0.01 (**) and 0.05 (*).

Treatment with cypermethrin resulted in reducing mean abundance of pre-adult and adult male stages (PAAM) as well as adult female stages of *L. salmonis* at all five sites (Table 1, Figure 1) which, according to univariate analysis indicates an effectiveness in excess of 90% for all sites.

**Table 1:** **Estimates and 95% CI of treatment effectiveness against all mobile stages of *L. salmonis *** **at five sites located in Western Norway according to the methods described in the text**

**Site**	**Interval of days after treatment**	**Estimate**	**95% CI**
A	11-15	99.82	99.20 - 99.99
B	13-16	100.00	99.48 - 100.00
C	7-11	98.46	97.00 - 99.34
D	13-16	93.32	88.36 - 96.57
E	8-13	95.14	90.70 - 97.84

**Figure 2:**
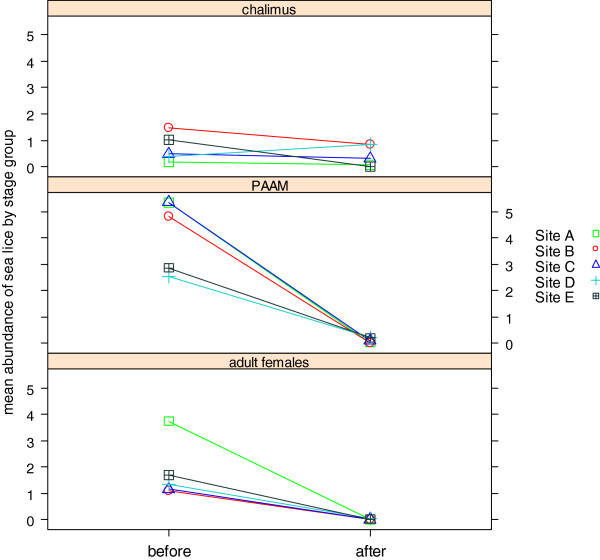
Values for abundance of chalimus, PAAM and adult females before and after treatment at each site.

The variation in mean abundance of the three stages between sites, shown in Figure 2, found the effect of treatment against PAAM and adult females was similar and consistent between sites but that the reduction in chalimus stages was modest with an average reduction in abundance of 49% (95% CI: 20–80). This value varied between sites, with one site indicating an increase in chalimus abundance following treatment. The reduction in chalimus was only significant at site E (100%), with sites B and C having less than 50% reduction. Tukey’s HSD test found that chalimus were significantly higher post-treatment (p-value <0.05) at sites B and D compared to A and E, and that site B was significantly higher than site C. Post-treatment pairwise site comparisons for PAAM and adult female stages indicated no significant differences.

### Changes in the composition of sea lice stages with treatment

**Figure 3:**
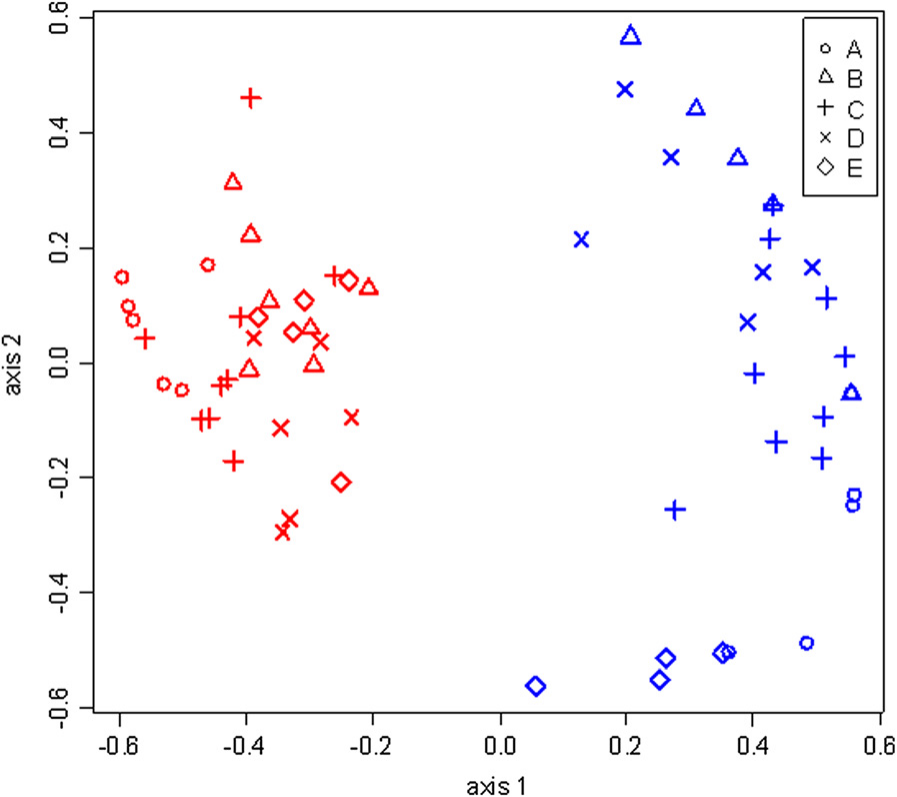
**Ordination diagram of the non-metric multidimensional analysis (NMDS) of abundance of *L. salmonis *stage groups.** Each symbol represents the abundance at each pen before (red) and after (blue) treatment at each site. The extent to which the symbols move after treatment reflects a change in composition of the three stages both per pen and per site. The low stress (0.13) and high correlation index (0.93) indicates concordance between plotted NMDS distances and distances based on abundance of sea lice between pens.

The changes in stage group composition from pre- to post- treatment were analysed using multivariate analyses. The effectiveness was assessed through the use of an ordination plot (stress = 1.3) (Figure 3) which provides a diagrammatic representation of the composition of stage groups at each site. The overlap of both pens and sites is shown in the clustering of pens before treatment (red symbols), whereas the dispersion of results across pens after treatment (shown in blue), indicated increased heterogeneity among pens and among sites. The within-site heterogeneity of stage groups both before and after treatment with cypermethrin is summarized in Table 2.

**Table 2:** Average and standard deviation (SD) of within-site distances, calculated from the matrix of Bray-Curtis distances both before and after treatment

**Site**	**Before**		**After**	
	**Average**	**SD**	**Average**	**SD**
A	0.148	0.071	0.328	0.170
B	0.241	0.120	0.306	0.202
C	0.271	0.161	0.315	0.134
D	0.207	0.095	0.357	0.134
E	0.219	0.114	0.302	0.176

Values close to zero and one are indicative of low and high dispersion, respectively.

Differences between sites both pre- and post- treatment, shown in Figure 2, were analysed by non-parametric permutational multivariate analyses (Table 3) to determine the extent to which production sites had similar composition of sea lice stages. Three procedures indicated statistically significant differences between sites in the composition of sea lice before (p-value <0.01) and after treatment (p-value <0.01).

**Table 3:** **Permutational multivariate analyses for the abundances of *L. salmonis *** **stage groups at five sites**

		**Before treatment**		**After treatment**	
**Multivariate approaches**	**Type of statistic**	**Value of statistic**	**p-value**	**Value of statistic**	**p-value**
ANOSIM*	R	0.3532	<0.005	0.5758	0.001
Adonis†	R^2^	0.45897	<0.005	0.62723	0.001
MRPP‡	A	0.2459	0.001	0.3589	0.001

The ANOSIM, Adonis and MRPP analysis calculated the statistic R, the R^2^ value and chance corrected within-group agreement (A), respectively, which indicated the magnitude of differences between group means.

*Analysis of similarities ANOSIM.

†Non-parametric multivariate analysis of variance (MANOVA) with the Adonis function.

‡A non-parametric procedure that does not assume normality for the distribution of data or homogeneous variances (1,000 permutations).

Results of the test statistics for MRPP (A statistic), ANOSIM (R statistic) and Adonis (R^2^ value) indicated that differences between sites became larger post-treatment (A = 0.359, R = 0.576, R^2^ = 0.627) than before treatment (A = 0.246, R = 0.353, R^2^ = 0.459) (Table 3). To identify which stage groups were responsible for the observed changes in composition between sites (p-value < 0.1, see Methods), we quantified the various life cycle stages (chalimus, PAAM and adult females) before and after treatment (Table 4). Prior to treatment, chalimus and adult females accounted for the differences in composition between sites; whereas after treatment the chalimus (p-value = 0.013) and PAAM (p-value = 0.066) stages were responsible. Following treatment, relative frequency and abundance of PAAM were higher at sites D and E; while for chalimus these were higher at sites B and D compared to all other sites. Counts of adult females were consistently low after treatment and had no significant indicator species value.

**Table 4:** **Frequency, abundance and indicator value (%) for the *L. salmonis *** **stage groups at five sites before and after treatment**

**A) Before treatment**								
**Stages**	**Statistic**	**Site A**	**Site B**	**Site C**	**Site D**	**Site E**	**Maximum site**	**p-value**
Chalimus	RF	0.166	1.000	0.667	1.000	1.000		
	RA	0.054	0.379	0.144	0.119	0.302		
	*IndVal*	0.009	0.379	0.096	0.119	0.302	B	0.049
PAAM	RF	1.000	1.000	1.000	1.000	1.000		
	RA	0.259	0.214	0.265	0.120	0.139		
	*IndVal*	0.259	0.214	0.265	0.120	0.139	C	0.405
Adult females	RF	1.000	1.000	1.000	1.000	1.000		
	RA	0.417	0.113	0.135	0.150	0.185		
	*IndVal*	0.417	0.113	0.135	0.150	0.185	A	0.001
**B) After treatment**								
Chalimus	RF	0.500	1.000	1.000	1.000	0.000		
	RA	0.033	0.432	0.154	0.381	0.000		
	*IndVal*	0.016	0.432	0.154	0.381	0.000	B	0.011
PAAM	RF	0.167	0.000	0.667	0.833	1.000		
	RA	0.029	0.000	0.172	0.411	0.387		
	*IndVal*	0.005	0.000	0.115	0.343	0.387	E	0.065
Adult females	RF	0.000	0.000	0.222	0.333	0.200		
	RA	0.000	0.000	0.148	0.584	0.267		
	*IndVal*	0.000	0.000	0.033	0.195	0.054	D	0.358

RF = Relative frequency, RA = Relative abundance, *IndVal* = Individual species values. Maximum site indicates the site that had the highest indicator value for each stage group. The p-value gives the probability of finding a higher indicator value in random permutations, with values of less than 0.1 indicating that the stage group is a significant factor.

At one site (site D) cypermethrin treatment was less effective against all stage groups. Analysis at pen level for site D showed that treatment against PAAM and adult females was effective for all pens (above 90%) except for one where the percentage reduction for PAAM was 63% (95% CI: 10–87) and for adult females was 82% (95% CI: 50–96).

## Discussion

Synthetic pyrethroids are widely used against sea lice in Norwegian aquaculture. Three synthetic pyrethroids are available for lice treatments, cypermethrin (Excis®) deltamethrin (Alpha Max®) and high-cis cypermethrin (Betamax®). These compounds have similar characteristics (administration and distribution), mechanisms of action, and therapeutic efficacies. Synthetic pyrethroids are insecticides that act by preventing closure of voltage-gated sodium channels resulting in abnormal hyper excitability, spastic paralysis and death. Synthetic pyrethroids are highly efficacious against PAAM and adult females [14] but reportedly less efficacious against chalimus stages [15,16].

Current analytical methods for evaluating treatment effectiveness calculate the percentage reduction in the mobile stages only. This method has low sensitivity when sampling is limited and is unlikely to fully reflect the treatment outcome [17] since calculation of treatment effectiveness is based on average abundance for only one stage group (PAAM or all mobiles). It is recognised that univariate analyses fail to control for experimental error and do not take account of the covariance structure in the data [18]. We performed multivariate analyses to combine the information from all group stages and determine the stage groups that best indicate (changes in magnitude or direction of) treatment effectiveness. This assessment of the community structure differs from the more statistical approaches to clustering adopted in previously published research [19,20].

In this study, we examined the effectiveness of cypermethrin treatments conducted in 33 pens at five different sites. Treatment with cypermethrin resulted in a larger than 90% reduction of all mobiles, which would conventionally be taken as an indication that treatment was effective at all sites. However, high treatment effectiveness against chalimus was observed only at one site, which suggests that chalimus was the only stage group that contributed to the significant differences in abundance observed between sites after treatment. Absolute numbers of chalimus were low which makes it difficult to evaluate treatment effectiveness. In addition these smaller stages are more difficult to enumerate accurately when sampling live fish in a production environment [21].

We had expected effective treatment to homogenize the initially heterogeneous sea lice populations, yet multivariate analysis and ordination analysis revealed that there existed a latent representation of sea lice stage composition that indicated increased heterogeneity following treatment. In particular chalimus and to a lesser extent PAAM were identified as stage groups accounting for the phenomenon observed.

Several site factors could offer explanations as to how differences in estimated treatment effectiveness arise. One factor may be water temperature. Low water temperatures can significantly delay the development of chalimus stages [22]. This is suggested by the limited reduction of chalimus in low temperatures at sites B and C, despite the significant reduction in PAAM. It has been shown that time to chalimus mortality following application of cypermethrin is increased with low temperatures [23,24]. The reduction in chalimus observed at site C was 37% (mean abundance was 0.31 [95% CI: 0.21-0.39]) 10 days after treatment; however, 20 days after treatment this reduction had increased to 90% (mean abundance at site C was 0.02 [95% CI: 0.00-0.06], while no increase in PAAM was observed), consistent with a delayed molting due to low temperature.

A further contributory factor may be the effect of cypermethrin on the development of the chalimus since cypermethrin delays metamorphosis within the chalimus stages [25]. The combination of temperature and developmental delay in chalimus will differentially affect the stage group composition and argues for the analysis of all stage groups when determining treatment effectiveness for this type of topical intervention [15,16]. Since univariate analysis utilizes only changes in PAAM or mobile populations the assessment of treatment effectiveness will be incomplete and potentially misleading.

The time interval between treatment and sampling was not identical across sites. The size of treatment effect will be influenced by the time interval between treatment and sampling. It may therefore be advisable to standardize the time at which treatment effectiveness is observed.

Using univariate methods, cypermethrin treatment was highly effective against PAAM stages, as defined by a reduction in the lice numbers equal or larger than 90%. Multivariate analyses questions this apparently satisfactory outcome. Effectiveness following treatment may not be as simple as a numerical reduction in a particular lice stage but may for example be a change in the distribution of population numbers across several lice cycle stages following treatment, an alteration of the sex ratio of the adults or an unforeseen delay in stage development. Multivariate methods yield data that may help more accurately define treatment effectiveness and we recommend the adoption of such methods in order to study empirical data from ‘best practice’ treatment studies.

Overall, cypermethrin treatment was effective at all production sites but differences were found in the composition of sea lice stages between sites after treatment. As treatment effectively reduced the PAAM and adult females this suggests that abiotic factors may account for differences in sea lice composition after treatment between sites. This aspect cannot be addressed from our data due to a limited sample size of five farms. Identification of these factors merits further investigation.

## Conclusions

The efficacy of outcome from topical treatment with synthetic pyrethroid is multifactorial, and not solely dependent on achieving adequate levels in the water column. With the emergence of chemical resistance there is a pressing need for comprehensive interpretation of collected data such as that offered by the multivariate approach. We have used multivariate methods to evaluate the effectiveness of cypermethrin treatment against sea lice. Multivariate methods, unlike the currently adopted univariate methods which focus on a single stage group of *L. salmonis*, provide an improved measure of the treatment effectiveness against all parasitic stage groups. Multivariate analyses could be extended to evaluate treatment against other ectoparasites of veterinary and medical importance.

## Methods

### Study area

The study included five Atlantic salmon (*Salmo salar*) sites (A, B, C, D and E) located in the Sogn and Fjordane county in the western region of Norway. Average seaway distances between sites were below 50 km, except for site E which was around 70 km from the nearest site. All bath treatments were conducted with full tarpaulin enclosures [21] using Betamax Vet® (Novartis Aqua Norge, Oslo, Norway) in accordance with manufacturer’s recommendations. Bath treatments were performed in all pens at each production site between November 2011 and February of 2012. The number of pens (p) per site varied from five to nine; for site A (p = 6), B (p = 7), C (p = 9), D (p = 6) and E (p = 5).

All treatments were completed within five days at a given site. Pens were treated consecutively at a rate of one or two pen treatments per day. Water temperatures (based on monthly average values at the time of treatment) were similar in three sites (C, D and E) ranging between 6.8 - 8.2°C. The coldest and warmest temperatures were recorded at site B (5.4°C) and site A (10.5°C), respectively. Delousing treatments in Norway are mandatory when lice levels exceed the thresholds provided in Norwegian regulations. As all pens on a site must be treated there is no opportunity to leave some pens untreated to act as controls, as would be possible in a clinical trial.

### Fish sampling

Sampling was performed before and after treatment at weekly or biweekly intervals. Pen samplings were conducted from ten days prior to, and up to approximately 50 days following treatment. All pens (n = 33) except one at site D (where fish were slaughtered) were sampled before and between 3 and 16 days after treatment; only two sites and around half of the pens were sampled after day 23. The total numbers of fish sampled were 455 before and 412 following treatment, respectively. Sample size per pen at each sampling point ranged from ten to 24 fish, with most groups comprising ten fish (62%). Each sample included a count of mobile *Caligus elongatus* and counts of *L. salmonis* for three lice cycle stages: chalimus, PAAM (pre-adult and adult males) and adult females. In this communication, we did not analyse data associated with *C. elongatus* as infestation with this species was only detected at one site before treatment and at very low levels. Counts of sea lice are routinely conducted by farmers and the results reported to authorities as mandated by Norwegian regulations [7]. Fish are sampled from each pen with a dip pen net, anesthetized for examination and returned to the pen after recovering from anaesthesia [26].

### Statistical analyses. Summary statistics before and after treatment

Arithmetic mean and median abundance (number of lice per fish) and prevalence (number of fish with lice) values were calculated for each stage group of *L. salmonis* to characterize the level of infestation at each site. For the analysis, we aggregated the count values for all pens within a site. Estimates and 95% confidence intervals were calculated using the adjusted bootstrap percentile (bias-corrected and accelerated, BCa) method [27] in the boot package in R [28,29]. We generated 1,000 bootstrap samples from the original data set. ANOVA was used to identify differences in the mean counts of *L. salmonis* between sites after treatment. When the effect of site was significant, we used a Tukey’s Honestly Significant Difference (HSD) to determine which sites differed from each other. All statistical analyses were carried out in Version 2.15.1 of R [30].

### Calculation of treatment effectiveness

Traditionally, treatment effectiveness is calculated as percentage reduction in PAAM or in all mobile stages (pre-adult and adult sea lice) [14]. We calculated treatment effectiveness based on counts taken approximately two weeks after treatment (between days 10 and 20 after treatment). Pre- post treatment comparisons are widely used to detect treatment effects and reflect efficacy. In particular in sea lice trials control pens are often unavailable and any observed lice reduction following treatment can be safely attributed to the treatment which has previously been widely demonstrated to be effective to obtain marketing authorisation. We calculated 95% confidence intervals using the quasi-Poisson method as this has been previously shown to be effective for this purpose [31,32].

### Multivariate analyses

The composition of *L. salmonis* stage groups was studied using Bray-Curtis distances in combination with non-metric multidimensional scaling (NMDS). These procedures are well suited for arthropod community analysis [33,34] since they avoid assumptions of linear relationships and are less susceptible to bias introduced by large numbers of zero counts in the data [35]. Bray-Curtis distances were calculated on untransformed data with the R package Vegan [36]. Guidelines for the interpretation of NMDS plots have been provided by Dufrêne [37]. Briefly, objects that are closer together within the NMDS plot are more similar (i.e. in terms of stage group composition) than those further apart. The stress value is used as a measure of goodness of fit between the original data (matrix of distances) and the ordered position of objects in the two dimensional space (NMDS configuration). Small stress values indicate a solution with good fit. In particular stress values below 0.1 indicate a good configuration, while values greater than 0.2 indicate a poor fit [38]. We did not calculate correlations between community dissimilarities and ordination distances, as this can be misleading when using a non-linear method (NMDS). Ordination using principal coordinate analysis produced similar results to those obtained with NMDS (data not shown).

In addition to ANOVA, we used three non-parametric procedures to statistically examine differences in the composition of *L. salmonis* between sites in response to treatment. Non-parametric procedures are preferred for data with skew distributions such as parasitic infestations. These procedures included the multiple response permutation procedure (MRPP), the analysis of similarities (ANOSIM) and permutational multivariate analysis of variance (Adonis). All these permutation procedures compared the ranks of distances between groups (farm sites) with the ranks of distances within groups. The site factor was tested in 1,000 permutations of residuals under the null hypothesis. To avoid finding falsely significant results, we performed an inferential statistical procedure similar to Levene’s test. This procedure is based on a permutation-based test of multivariate homogeneity of group dispersions (variance in the sites) [39]. Results from inferential testing indicated that the within-group dispersion was not significantly different between sites before and after treatment (data not shown) [40].

The MRPP tests the relationship of entities in the multidimensional space by comparing the weighted mean of within-site distances to the within-site means from randomly assigned sites. A significant p-value (<0.05) indicates that differences detected between sites are greater than would be expected from random assignment to sites. It also provides a measure of the magnitude of differences between group means (A); computed as A = 1- (δ/m_δ_), where the observed delta (δ) describes the weighted mean within-site distance, and the expected delta (m_δ_) is computed as the mean delta for all possible partitions of the data. For example, when the composition of sea lice at pens within-sites are identical, then δ = 0 and A = 1. The value of A becomes smaller as the level of agreement is increasingly reduced from than that expected by chance. The advantage of the MRPP statistic is that it is robust to unequal variance, non-normally distributed data and unbalance designs [34].

Analysis of similarities (ANOSIM) [12] is similar in concept to MRPP but uses a different test statistic. The result is summarized in the R statistic which indicates the magnitude of difference between group means. The R statistic ranges from 0 (no separation) to 1 (high separation). R values >0.75 are indicative of high separation, R >0.5 as separated but overlapping and R <0.25 as barely separable.

The permutation multivariate analysis of variance PERMANOVA (Adonis) is a permutation-based version of the multivariate analysis of variance [41]. Similar to the other permutation tests, it uses distances between sites to partition variance. Significance testing is carried out using F-tests derived from permutations of the raw data.

### Indicator species analysis

The concept of indicator species has been previously used in the fields of marine ecology [42-44]. For this Dufrêne and Legrende [37] proposed a flexible and asymmetrical approach to identify indicator species. This method combines the relative abundance (specificity) with the frequency (fidelity) of species (or stage groups) at a site and finds the stage groups that are significantly concentrated at a site or group of sites. Stage groups with significant indicator species values provide some measure of the characteristic of a site and can be used to monitor changes.

For each stage group *i* in each pen of site *j*, the relative abundance *RA*_*ij*_ and the relative frequency are computed as *RF*_*ij*_:

$$RA_{\mathrm{ij}}=\frac{A_{\mathrm{ij}}}{A_{i}}.$$

where *A*_*ij*_ is the mean abundance of stage group *i* across pens of the site *j*, *A*_*i.*_ is the sum of mean abundances of stage group *i* over all sites.

$$RF_{\mathrm{ij}}=\frac{S_{\mathrm{ij}}}{S_{.j}}$$

where *S*_*ij*_ is the number of pens in site *j* where stage group *i* is present, *S*_*.j*_ is the total number of pens in that site. Combining the relative abundance and frequency gives the indicator species value of stage group *i* at site *j*

$$\mathrm{IndVal}=RA_{\mathrm{ij}}\times RF_{\mathrm{ij}}$$

A stage group may be considered characteristic of a site if it has an *IndVal* value greater than 25% and a p-value < 0.1, as discussed in [36]. The significance level was increased to decrease the Type II error that is commonly found as a result of low power resulting from the permutation test with a low number of replicates (pens). The p-value for a Monte Carlo test (1,000 permutations) evaluates the statistical significance of the *IndVal*.

## Competing interests

The authors declare that they no competing interests.

## Authors’ contributions

GG, CWR, DFJ conceived and designed the study. DFJ performed the statistical analysis. All authors contributed in the writing of the paper. All authors read and approve the final manuscript.
